# Supplementary figures and images for: Isolation and characterization of lumpy skin disease virus from cattle in India
Source: PLoS One. 2021 Jan 11;16(1):e0241022. doi: 10.1371/journal.pone.0241022 (PMC7799759; doi:10.1371/journal.pone.0241022)

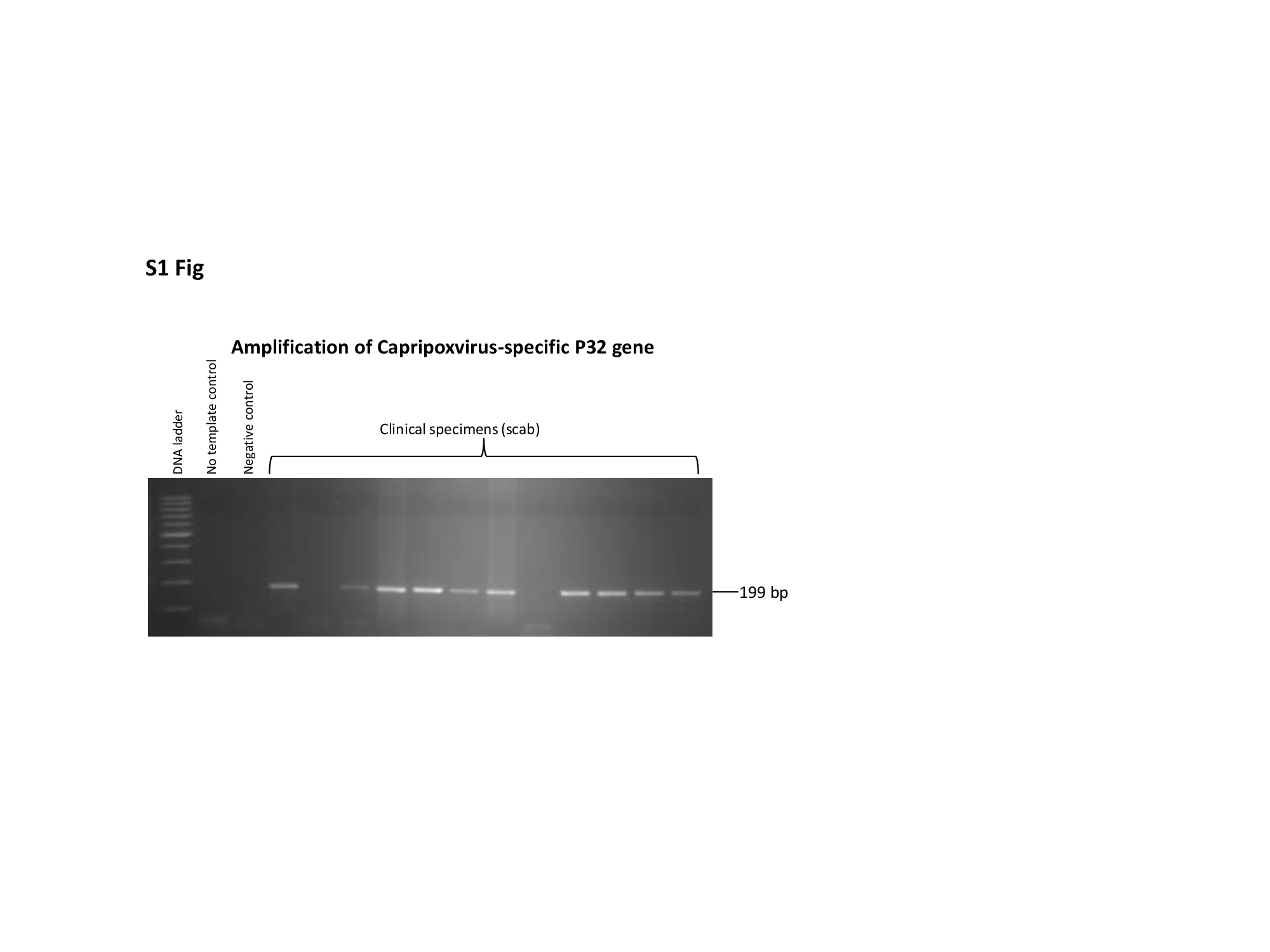

Supplement: S1 Fig — Virus was recovered from the scabs in DMEM followed by DNA extraction and PCR to amplify capripoxvirus-specific P32 gene. (TIFF) [file pone.0241022.s001.tiff]

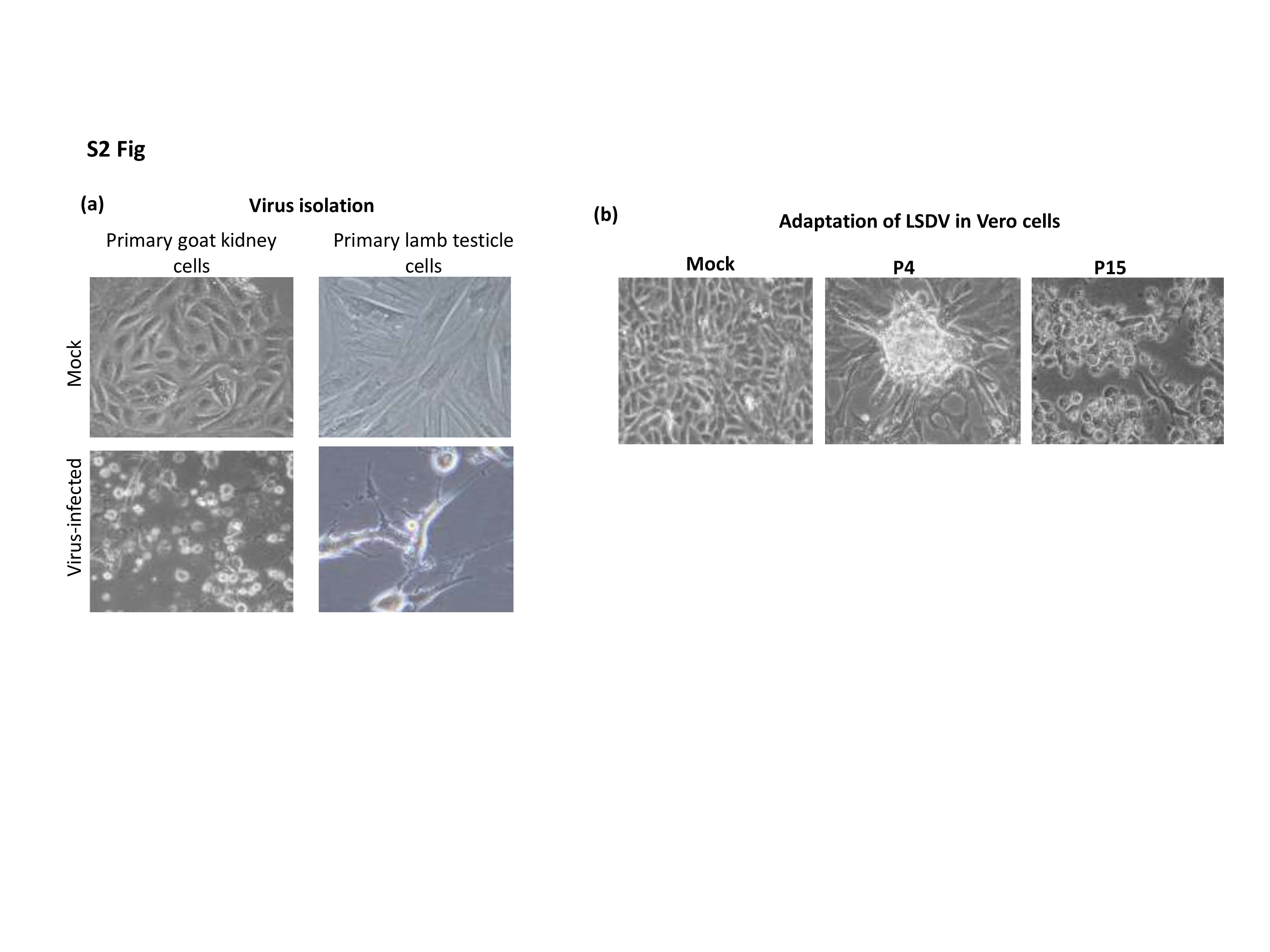

Supplement: S2 Fig — Virus isolation (a) Virus isolation. An aliquot of the virus (500 μl filtrate) was used to infect confluent monolayer of PGT, PGK and PLT cells for 2 h followed by addition of fresh growth medium. The cells were observed daily for appearance of the CPE. The CPE observed in PGK, PLT and MDBK cells is shown. (b) Adaptation to Vero cells. An aliquot (500 μl) of the LSDV isolated in PGK cells was used to infect confluent monolayers of Vero cells for 2 h followed by followed by addition of fresh growth medium without serum. The cells were observed daily for appearance of CPE. The CPE observed at P4 and P15 is shown. (TIFF) [file pone.0241022.s002.tiff]

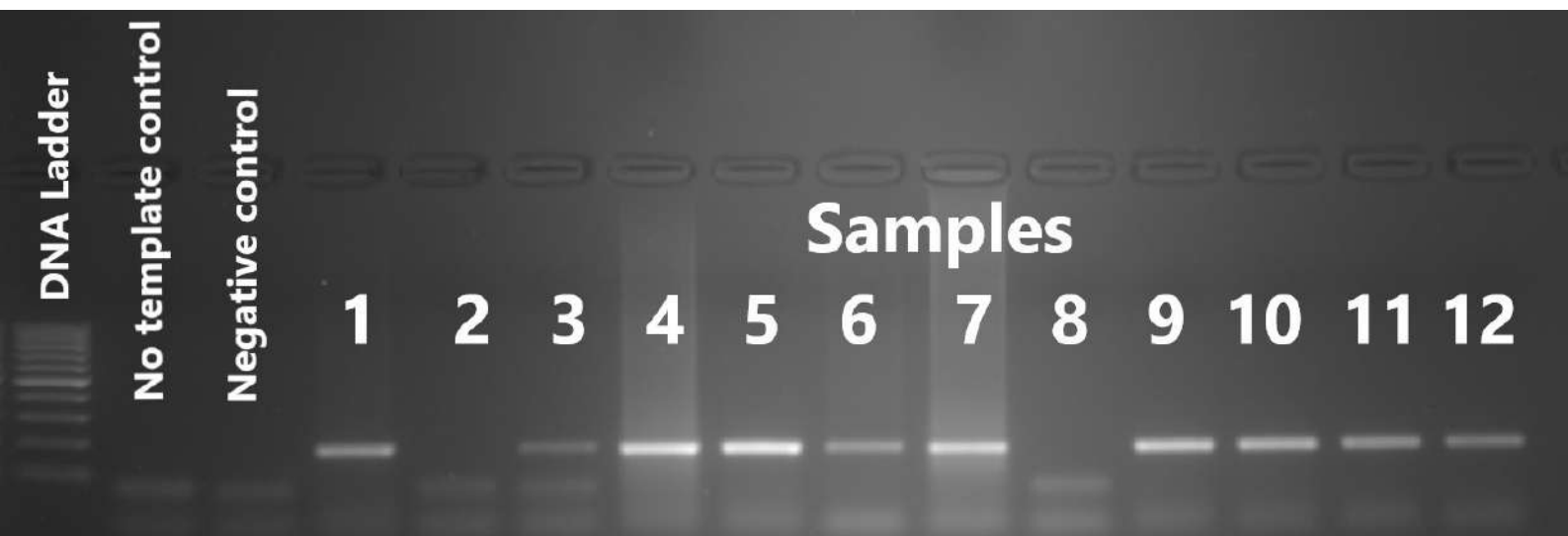

**S1 Fig\_Raw\_Image**

Supplement: S1 Raw image — (PDF) [file pone.0241022.s003.pdf]
