# Supplementary material for: Isolation and characterization of lumpy skin disease virus from cattle in India
Source: PLoS One. 2021 Jan 11;16(1):e0241022. doi: 10.1371/journal.pone.0241022 (PMC7799759; doi:10.1371/journal.pone.0241022)
Supplement: Attachment — Submitted filename: Responswes to the Reviewers comments.doc [file pone.0241022.s004.doc]

**Responses to the Reviewer’s comments**

**Reviewer #1:**

The authors describe the first successful isolation of LSDV from a natural outbreak in India. LSDV as causative agent was confirmed by clinical observation, PCR, partial sequencing and serum neutralization assay. In addition, three different primary cell lines were examined regarding their susceptibility towards LSDV, and utilization of an alternative permanent cell line (Vero cells) for propagation of LSDV was analyzed. Moreover, authors compared viral genome load and virus titer in cell culture systems at different time points post infection to gain insight in the life cycle of LSDV.

General
• l. 184: phosphate buffered saline

**Corrected as suggested (*Line 185 in the revised manuscript)***

• l. 211: subcutis

**Corrected as suggested *(Line 213 in the revised manuscript).***

• l. 236: LSDV/SPV/GPV

**Corrected as suggested *(Line 243 in the revised manuscript).***

• l. 255: PGT

**Corrected as suggested *(Line 262 in the revised manuscript).***

• l. 256: Why the MDBK cells were not used for virus isolation in comparison?

**Primary cells of ovine, caprine and bovine origin are considered to be the most sensitive cells for LSDV isolation . MDBK cells have been used to propagate the LSDV at high titres . Some studies also suggest use of MDBK cells for LSDV isolation . However, it is uncertain whether these are more sensitive than primary cells for LSDV isolation. We had primary goat kidney cells, primary lamb kidney cells and primary lamb testicle cells already in hand, so it’s not worth employing MDBK cells for virus isolation. However, later (as per the suggestions of the Reviewer), we also infected the MDBK cells with the clinical specimen(s) but CPE could not be observed at the first passage as it was observed in PGK (primary goat kidney) cells. This suggests that MDBK cells may not be as sensitive as PGK cells for virus isolation. However, we believe that this cannot be used as proof of principle as virus isolation from clinical specimens may be influenced by several factors such as (i) virus titre in the clinical specimens (ii) nature of cryptic (unknown) agents present in the cell culture system (iii) co infecting (unknown) agents present in the clinical specimens.**

• l. 257: brackets should be deleted

**Corrected as suggested *(Line 256 in the revised manuscript).***

• l. 295: maybe better “Serum samples from all the clinically affected animals that showed presence of…” instead of brackets

**Corrected as suggested *(Line 235-236 in the revised manuscript).***

• l. 298: titer of 1:64 to 1:1024

**Corrected as suggested *(Line 238 in the revised manuscript).***

• l. 308: “from India in 2019”

**Corrected as suggested *(Line 314 in the revised manuscript).***

• l. 311: “(until this paper is being written)” is not necessary

**Corrected as suggested**

• l. 352: TCID50

**Corrected as suggested *(Line 362 in the revised manuscript).***

• l. 379: phase be consistent with spelling “titer” (AE) or titre (BE) during the manuscript
**“Titre” has been followed in the entire manuscript**

Figures and Tables

• Figure 2: brackets not necessary

**Figure 2 has been deleted in the revised manuscript**

• Figure 3: could be given as supplemental information

**Figure 3 has been shifted to supplementary information *(S1 Fig in the revised manuscript)***

• Figure 5: cold be given as supplemental information

**Figure 5 has been shifted to Supplemental information *(S2 Fig in the revised manuscript)***

• Table 2

o footnotes (*, #) should be described

**Foot notes have been described *(Line 232-233 in the revised manuscript).***

o information about how many scabs were taken from an individual and how many scabs were taken at the same farm would be helpful

**At least 2 scabs were taken from each individual animal. The number of animals from an individual farm varies, depending on the availability of the affected animals. Please see Table 2 *(Line 232-233 in the revised manuscript).***

o please specify scab 22 “1 month later”, is this related to an individual animal/farm (which one?) or in general?

**This is in relation to the individual animal (serial number 15). It has been mentioned in the Table 2 accordingly *(Line 232-233 in the revised manuscript).***

o In place of Viremia, better: LSDV genome (blood)

**Viremia replaced with LSDV genome (blood) *(Line 232-233 in the revised manuscript).***

o Antibody titer

**Corrected as suggested *(Line 232-233 in the revised manuscript).***

o Antibody titer should be indicated as 1:xx or transferred into ND50/ml

**Corrected as suggested (written as 1:XX) *(Line 232-233 in the revised manuscript).***

• Figure legend Figure 2: explain red triangles

**Figure 2 has been deleted in the revised manuscript**

Results
• Structure outbreak and later cell culture work together – in detail:

**Structured as suggested**

o “reactivity of LSDV to the sera from LSDV-infected animals” should be between “Identification of the agent” and “phylogenetic analysis” as it belongs to the examination of the outbreak and results are additionally presented in Table 2

**Rearranged the text as per the suggestions**

**Reviewer #2:**

What are the main claims of the paper and how significant are they for the discipline?

• The paper describes outbreaks of LSDV in India, and contains interesting details such as the existence of serologically positive animals which display no clinical signs, morbidity data, and phylogenetic analyses of the virus isolated. This information will be important to researchers in the field and policy makers who are trying to control the current LSD epidemic in Asia.

**We thank the Reviewer for his/her enthusiasm towards this study.**

Are the claims properly placed in the context of the previous literature? Have the authors treated the literature fairly?

• The literature is cited well, and most of the claims are properly placed in the context. However the utility and widespread use of MDBK cells to isolate and analyse capripoxviruses is not highlighted sufficiently.

**Use of MDBK cells for LSDV isolation and propagation has been described in detail in the revised manuscript. *(Line 349-362 and 368-371 in the revised manuscript).***

Do the data and analyses fully support the claims? If not, what other evidence is required?

• The majority of the claims are well supported. The only unsupported claim is the use of Vero cells for the propagation of LSDV (line 367-370 and elsewhere). The authors clearly show that Vero cells cannot support propagation of field strains of LSDV (line 264-269). The virus had to be passaged 15 times in order for CPE and viral plaques to be detected on Vero cells. The literature, however, shows that MDBK cells do not require such virus adaptation and CPE can be detected on first passage.

The authors should therefore have concluded that Vero cells are not permissive for LSDV isolation and are inferior to MDBK cells in this respect. Their conclusion that Vero cells are suitable for the propagation of LSDV is incorrect and not supported by their results.

**We thank the Reviewer for this important suggestion. In fact we realised that some of our conclusions about the Vero cells were somewhat overinterpreted. In the revised manuscript, comparison on Vero and MDBK cell culture has been elaborated and some of the over interpretations made about Vero cells have been removed/rewritten. Also the suitability of MDBK cells for LSDV isolation and propagation has been discussed in detail. *(Line 382-385 and 297-206 in the revised manuscript).***

• Figure 5C is very indistinct, can the authors provide a better quality one? The plaques are not distinguishable on the current figure

**Our main aim of propagating the LSDV in Vero cells was to produce plaques. Unlike the primary cells, Vero-adapted LSDV produced plaques but were of small size to capture a good image. Therefore, in the revised manuscript, we have removed Fig 5c.**

• Please provide statistical analyses for the comparisons between Vero cells and MDBK cells in Figure 6. Are the differences reported statistically significant?

**We have provided statistical analysis wherever applicable. The differences in the viral titres between Vero and MDBK cells are apparently significant. However, the statistical calculation is only possible at certain time points (96hpi, 144 hpi and 196hpi) wherever there are variations in the biological replicates. The absence of any variations in three the biological replicates limits the calculation of significant statistical difference.**

**However, we have provided the statistical analysis for the levels of viral DNA in Vero and MDBK cells at different time points. Pair-wise statistical comparisons were performed using Student’s t test. *(Fig 3 and Line 542-548 in the revised manuscript).***

• Please re-examine the legend for Figure 6. It is not correct. A and B are growth curves. C and D (not currently mentioned in the legend) are PCRs.

**We thank the Reviewer for pointing out this mistake. The legend has been corrected *(Line 528-534 in the revised manuscript).***

• Make it clear in the legend and text for Figure 6 that the data presented is for the cell culture adapted strain of LSDV, not a field strain. This is very important for diagnosticians who may read the manuscript and not realise straight away that Vero cells do not support LSDV isolation.

**We have mentioned in the Figure legend and discussion section that this is applicable to cell culture adapted strain of LSDV *(Line 190, 378, 388 and 529 in the revised manuscript).***

• Do not use PFU/ml as a synonym for TCID50, as described in the legend of Figure 6. They are very different things. If you counted plaques (or foci) then use PFU. If you examined cells for CPE, use TCID50.

**We thank the Reviewer for pointing out this issue. Virus titres were determined by TCID_50_ assay and converted to estimated plaque-forming units (PFU) by the conversion TCID_50_ ≈ 0.7 PFU as per the standard conversion formula described previously by Coves-Datson EM et al (2020, PNAS, USA). This was carried out to ensure homogeneity in the expression of viral titres (PFU/ml or TCID_50_/ml).**

PLOS ONE encourages authors to publish detailed protocols and algorithms as supporting information online. Do any particular methods used in the manuscript warrant such treatment? If a protocol is already provided, for example for a randomized controlled trial, are there any important deviations from it? If so, have the authors explained adequately why the deviations occurred?

• Not applicable

**We thank the Reviewer**

If the paper is considered unsuitable for publication in its present form, does the study itself show sufficient potential that the authors should be encouraged to resubmit a revised version?

• Not applicable

**We thank the Reviewer**

Are original data deposited in appropriate repositories and accession/version numbers provided for genes, proteins, mutants, diseases, etc.?

• The LSDV strain has been deposited in a repository (lines 261-262) although this could not be confirmed on the website of the depository

**We have asked the concerned repository to update the information of our deposited LSDV strain. In fact the information has been updated. Please visit at** [**www.ncvtc.org.in**](http://www.ncvtc.org.in/) **(Under Distribution of Microbes)**

Are details of the methodology sufficient to allow the experiments to be reproduced?
• Yes

**We thank the Reviewer**

Is the manuscript well organized and written clearly enough to be accessible to non-specialists?

• Yes, it is very nicely written.

**We thank the Reviewer**
