# Supplementary material for: Isolation and characterization of lumpy skin disease virus from cattle in India
Source: PLoS One. 2021 Jan 11;16(1):e0241022. doi: 10.1371/journal.pone.0241022 (PMC7799759; doi:10.1371/journal.pone.0241022)
Supplement: Attachment — Submitted filename: R2 Responswes to the Reviewers comments.doc [file pone.0241022.s005.doc]

**Responses to the Reviewer’s comments**

**Reviewer #1:**

The performed changes in the revised manuscript are fine for me. This revised version is acceptable for publication.

**We thank the Reviewer for considering our manuscript acceptable for publication**

**Reviewer #2:**

The authors have addressed the majority of the comments by the reviewers. However they need to make very clear throughout the manuscript that they are analysing their laboratory adapted strain of LSDV in figure 3, and not a wild-type strain.

**We have mentioned “Vero cell-adapted LSDV” instead of LSDV wherever it’s required (please see line number 35, 40, 190, 292, 295, 296, 388, 396, 398, 528-529 in the revised manuscript).**

The inability of Vero cells to support propagation of LSDV needs to be clearly stated.

**Even in the previous version of the manuscript we had clearly mentioned (Line number 382-385 in the current and previous version of the manuscript) that Vero cells are not suitable for LSDV isolation from the clinical specimens.**

The authors also need to make clear that MDBK cells are a suitable and widely used cell line for LSDV isolation and propagation. This information is very important for diagnosticians who may read the manuscript and not realise straight away that Vero cells do not support LSDV isolation. Thus please take in account the comments from the reviewer 2.

**In the previous version of the manuscript, we had already described (Line number 357-361 in the current and previous version of the manuscript) that MDBK cells are also sensitive for LSDV isolation. We never compared Vero cells with MDBK/primary cells for virus isolation. We just compared the growth of Vero cell-adapted LSDV in Vero and MDBK cells (Fig. 3) where Vero cells were certainly found better than MDBK cells for the growth of LSDV (Vero cell-adapted).**

Please correct “LSDV” to “Vero-cell adapted strain of LSDV” on line 35, 39, 292, 295, 298, 302, 394, 398 (twice) and 536. Also in the legend to Figure 3.

**We have mentioned “Vero cell-adapted LSDV” instead of LSDV wherever it’s required (please see line number 35, 40, 190, 292, 295, 296, 388, 396, 398, 528-529 in the revised manuscript).**

Add MDBK cells to the list of cells used for LSDV isolation on line 349-350.

**Again, in the previous version of the manuscript, we had already described (Line number 357-361 in the current and previous version of the manuscript) that MDBK cells may also be used for LSDV isolation.**

Delete lines 379-380 and 396 which state that Vero cells are suitable for the propagation of LSDV. They are clearly not.

**We totally disagree with the Reviewer about deleting this statement because this is about the growth of Vero cell-adapted LSDV (not wild type LSDV) where Vero cells produced higher titer than MDBK cells.**

Line 95-96 Delete the statement “However, a plaque assay to precisely quantify infectious LSDV is still lacking.” This plaque assay has been described in Fay et al 2020.

**The paper by Beard’s group (Fay et al., 2020) describes focus forming assay. Unlike plaque assay, the foci (formed in this assay) need to be counted under microscope, thereby it is not considered as good as plaque assay where plaques can be directly counted with naked eyes.**

Figure S2 (b) the images are out of focus, which precludes examination of the CPE at p15. Do the authors have a better image?

**We have provided a different better image**

Figure S2 (b) LSDV is spelt incorrectly in the figure heading.

**LSDV spell our correctly**
